# Association Between Relative Cord Hyperleptinemia and Offspring Outcomes in Pregnant Women With Overweight or Obesity

**DOI:** 10.1111/ijpo.70144

**Published:** 2026-08-21

**Authors:** Jincy Immanuel, Stephy Chirayath, Jürgen Harreiter, Gernot Desoye, Rosa Corcoy, Juan M. Adelantado, Roland Devlieger, Annunziata Lapolla, Maria G. Dalfra, Alessandra Bertolotto, Ewa Wender–Ozegowska, Agnieszka Zawiejska, Fidelma P. Dunne, Peter Damm, Elisabeth R. Mathiesen, Dorte M. Jensen, Lise Lotte T. Andersen, Mette H. Tanvig, David J. Hill, Judith G. M. Jelsma, Alexandra Kautzky–Willer, Sander Galjaard, Frank J. Snoek, Mireille N. M. van Poppel, David Simmons

**Affiliations:** ^1^ School of Medicine Western Sydney University Sydney Australia; ^2^ College of Nursing Texas Woman's University Denton Texas USA; ^3^ Department of Medicine III, Division of Endocrinology, Gender Medicine Unit Medical University of Vienna Vienna Austria; ^4^ Department of Medicine and Palliative Care Landesklinikum Scheibbs Scheibbs Austria; ^5^ Department of Obstetrics and Gynecology Medizinische Universitaet Graz Graz Austria; ^6^ Department of Medicine Universitat Autònoma de Barcelona Barcelona Spain; ^7^ Institut de Recerca de L'hospital de la Santa Creu i Sant Pau Barcelona Spain; ^8^ CIBER Bioengineering, Biomaterials and Nanotechnology Instituto de Salud Carlos III Madrid Spain; ^9^ REALIFE Research Group, Department of Development and Regeneration KU Leuven Leuven Belgium; ^10^ Department of Obstetrics and Gynaecology University Hospitals Leuven Leuven Belgium; ^11^ Universita Degli Studi di Padova Padua Italy; ^12^ Azienda Ospedaliero–Universitaria Pisana Pisa Italy; ^13^ Department of Reproduction Poznan University of Medical Sciences Poznan Poland; ^14^ National University of Ireland Galway Ireland; ^15^ Center for Pregnant Women With Diabetes, Departments of Endocrinology and Obstetrics Rigshospitalet Copenhagen Denmark; ^16^ Department of Clinical Medicine University of Copenhagen Copenhagen Denmark; ^17^ Steno Diabetes Center Odense Odense University Hospital Odense Denmark; ^18^ Department of Gynecology and Obstetrics Odense University Hospital Odense Denmark; ^19^ Department of Clinical Research, Faculty of Health Science University of Southern Denmark Odense Denmark; ^20^ Lawson Research Institute London Ontario Canada; ^21^ Amsterdam UMC Vrije Universiteit Amsterdam, Department of Public and Occupational Health, Amsterdam Public Health Research Institute Amsterdam the Netherlands; ^22^ Department of Obstetrics and Gynaecology, Division of Obstetrics and Prenatal Medicine, Erasmus MC University Medical Centre Rotterdam the Netherlands; ^23^ Amsterdam UMC Vrije Universiteit Amsterdam, Department of Medical Psychology Amsterdam the Netherlands; ^24^ Department of Human Movement Science, Sport and Health University of Graz Graz Austria

**Keywords:** childhood adiposity, cord leptin, fat mass, pregnancy

## Abstract

**Background/Objectives:**

Umbilical cord leptin is increased in infants of women with obesity. We tested the hypothesis that a high cord leptin‐to‐fat mass ratio might be associated with adverse outcomes at birth and in early childhood.

**Methods:**

A secondary analysis of vitamin *D A* nd *L* ifestyle *I* ntervention for gestational diabetes mellitus prevention (DALI) trial was conducted. Cord leptin‐to‐fat mass ratios were classified into top (TT), middle, and low (LT) tertiles overall and within sexes. Relationship with neonatal outcomes and offspring BMI *z*‐scores were compared between TT and LT groups.

**Results:**

Among 323 infants (maternal pre‐pregnancy BMI 33.7 ± 4.2 kg/m^2^, 52% male) female (vs. male) offspring had higher cord leptin, fat mass percentage, and leptin‐to‐fat mass ratio (median (IQR): 21.4 (13.4, 30.99) vs. 15.5 (8.3, 25.9), *p* < 0.001). Compared to the LT group, infants in the TT group (58% females) had higher cord leptin but similar fat mass percentage. Among males (but not in females), the C‐peptide‐to‐glucose ratio was elevated in the TT group (vs. LT) (0.2 (0.1–0.2) vs. 0.1(0.02–0.2), *p* < 0.001), while birthweight (3.5 ± 0.5 vs. 3.7 ± 0.4 kg, *p* = 0.045), fat mass percentage (11.1 ± 3.9 vs. 13.0 ± 3.6, *p* = 0.008), and BMI *z*‐scores (at age 1 only) were lower (median (IQR): −0.15 (−0.67, 0.98) vs. 1.35 (−0.06, 1.67), *p* = 0.03).

**Conclusions:**

Relative cord hyperleptinaemia was associated with reduced offspring BMI among males.

**Abbreviations**
ANOVA: Analysis of variance
APGAR: Appearance, pulse, grimace, activity, respiration
BMI: Body mass index
CI: Confidence interval
DALI: Vitamin D and Lifestyle Intervention for gestational diabetes mellitus prevention
ELISA: *Enzyme‐linked immunosorbent assay*
GDM: Gestational diabetes mellitus
HAPO: Hyperglycemia and adverse pregnancy outcome
HAPOFUS: Hyperglycemia and adverse pregnancy outcome follow‐up study
HE: Healthy eating
HMW: High molecular weight
IADPSG: International Association of Diabetes in Pregnancy Study Group
IQR: Interquartile range
LT: Low tertile
MT: Middle tertile
NICU: Neonatal intensive care unit
PA: Physical activity
PIH: Pregnancy induced hypertension
SD: Standard deviation
TT: Top tertile

## 1 Introduction

Maternal overweight and obesity have become major concerns worldwide; they affect almost half the pregnancies in the United States and are projected to affect one in four pregnancies globally by 2030 [1]. Evidence has shown that exposure to an obesogenic intrauterine environment could predispose infants to overweight and obesity, particularly in late childhood [2, 3]. Recent data indicate that obesity affects up to 20% of children and adolescents [4]. Approximately 42% of this childhood adiposity is attributed to an increased maternal pre‐pregnancy body mass index (BMI) [2]. Possible mechanisms for offspring overgrowth found in a maternal obesity or an obesogenic diet, aside from any associated dysglycaemia, include altered nutritional transfer, upregulation of amino acids and glucose transport, decreased beta cell mass, and adipocyte hypertrophy. In addition, increased orexigenic neuropeptides from neuroepithelial differentiation and translocation can lead to hyperphagia and weight gain [5]. Animal models have shown high leptin concentrations and weakened leptin signalling in the hypothalamus, leading to leptin resistance and hyperphagia [5].

Increased concentrations of leptin in both maternal and fetal blood have been found among mothers with overweight or obesity compared with normal weight mothers [6, 7]. Several studies have examined the association of cord blood leptin with the neonatal, childhood, and pubertal adiposity using different adiposity measurements [8, 9, 10, 11]. Findings varied depending on age, showing a positive association with adiposity at birth and 11 years [8, 9, 10]; an inverse relationship with adiposity until 3 years [9, 10]; and no association at 4,5,7, and 17 years [9, 11]. The relationship also differed by sex with the strongest associations in males [10, 12], though the results were inconsistent across studies [10, 12, 13]. Moreover, all these studies focused on the absolute concentration of leptin and did not take into account differences in fat mass.

Infants born to mothers with obesity are a unique population group at increased risk of developing insulin resistance in utero [14] and possibly leptin resistance. Therefore, the purpose of this study was to test whether infants born to mothers with overweight or obesity who were enrolled in the vitamin *D A*nd *L*ifestyle *I*ntervention for gestational diabetes mellitus prevention (DALI) study would be leptin resistant at birth and have adverse outcomes as a result. In addition, we explored whether the lifestyle interventions mothers received during the study or their gestational weight gain influenced this relationship. Leptin resistance was assessed based upon the leptin‐to‐fat mass ratio as performed in past leptin studies [15, 16], where it accounted for differences in fat mass. Our hypothesis was that a higher cord leptin‐to‐fat mass ratio (relative cord hyperleptinemia) in pregnant women with overweight or obesity, putatively reflecting ‘leptin resistance’, might be associated with adverse pregnancy outcomes and adiposity in childhood.

## 2 Materials and Methods

A secondary analysis was performed using data from the vitamin *D A*nd *L*ifestyle *I*ntervention for gestational diabetes mellitus prevention (DALI) trial (ISRCTN70595832). The DALI protocol and the main findings from the study have been published elsewhere [17, 18]. In brief, the DALI trial investigated whether healthy eating, physical activity, and vitamin D supplementation, alone or in combination, prevented the development of gestational diabetes mellitus (GDM) in women with overweight or obesity. Singleton pregnant women aged ≥ 18 years with a BMI above 29 kg/m^2^ were recruited before 19^+6^ weeks of gestation from 9 European countries between 2012 and 2015. Women with known diabetes, major medical or psychiatric conditions, a diagnosis of GDM before 20 weeks, or those who were unable to communicate with lifestyle coaches or follow exercise or diet instructions were excluded from the study. Eligible women were randomly assigned to 8 different arms (healthy eating (HE), physical activity (PA), vitamin D, placebo, HE + PA, HE + PA + vitamin D, HE + PA + placebo, and control) stratified by site [17]. None of these interventions were found to be effective in improving fasting glucose or insulin resistance [18]. Data from the whole DALI cohort were used in the current analysis which included the DALI pilot study, the main trial and the vitamin D trial.

### 2.1 Data Collection

Data collection took place at < 20 weeks, 24–28 weeks, 35–37 weeks, and birth and < 48 h postnatal. Pre‐pregnancy weight was self‐reported. An oral glucose tolerance test was performed at the first three study visits, and those diagnosed with International Association of Diabetes in Pregnancy Study Group (IADPSG) criteria defined GDM managed as per the local guidelines. Maternal laboratory investigations, including leptin, adiponectin, glucose, insulin, and lipids were conducted at all time points except at delivery. The HMW Human Adiponectin samples were measured using the Quantikine immunoassay (R & D ELISA assay system, sensitivity: 0.989 ng/mL, intra‐assay precision: 2.6%–3.7%, and inter‐assay precision: 8.3%–8.6%). The samples were measured over 7 separate assays, each using a 96 well plate. Each assay had internal control value repeats for internal consistency for the low, middle and high sections of the assay curves. The intra‐assay variability from these replicate plates was between 5% and 8%.

### 2.2 Neonatal Assessment

Venous blood from placental chorionic vessels was collected and processed within 30 min after birth. The samples were centrifuged and the partitioned aliquots, 1 mL or 0.25 mL, were placed in microrack tubes and stored at −20°C or −80°C for further analysis in the central laboratory in Graz. Leptin concentrations were quantified by solid‐phase sandwich ELISA (E05–086‐96; EIASON, Graz, Austria) according to the manufacturer's instructions. The analytical sensitivity of the ELISA assay was 1.0 ng/mL, and the intra‐ and inter‐assay coefficients of variation (low/high concentrations) were 6.0%/6.9% and 11.6%/8.7%, respectively.

Neonatal measurements were performed within 48 h after birth. Assessment included weight, length, head circumference, anthropometry assessment, and skin‐fold measurements (triceps, quadriceps, subscapular, and suprailiac). Skin‐fold measurements were performed using the Harpenden calliper as described previously [17]. Each measurement was duplicated, and the average was recorded. If the difference between the two measurements was more than 0.2 mm, a third measurement was taken.

### 2.3 Outcome Measures

The main outcome measures of this study were childhood BMI and cord serum C‐peptide, cord serum glucose, C‐peptide‐to‐glucose ratio, Apgar score, hyperbilirubinemia, large and small for gestational age, and neonatal intensive care unit (NICU) stay. An Apgar score was taken at 3 and 5 min after birth. Complications before birth including pregnancy induced hypertension (PIH) and preeclampsia, as well as preterm birth and caesarean delivery, were also reported. Childhood BMI was calculated using available height and weight follow‐up data from surveys given to DALI participants who were contacted annually for offspring weight and height over 5 years.

Written informed consent was obtained from all study participants. The trial received approval from the following ethical committees: NRES Committee East of England—Norfolk: 11/EE/0221, Medical University Poznan: 1165/12, UZ KU Leuven: ML7625, VUmc Amsterdam: 2012/400, Hospital De La Santa Creu i Sant Pau Barcelona: 13/006 (OBS), Medical University Vienna: 2022/2012–1369/2013, Region Hovedstaden Copenhagen: H‐4‐2013‐005, Province of Padua: 4201 × 11, and Galway University Hospitals: 7/12 [14]. Approval for database hosting was approved by the Human Research Ethics Committee, Western University, London, Ontario.

### 2.4 Statistical Analysis

The *z*‐score for birth weight adjusted for sex and gestational age, and the *z*‐scores for BMI from ages 1 through age 5 were estimated using the Fenton growth chart [19] and the World Health Organization tool [20], respectively. Neonatal fat mass was calculated using the Deierlein formula: −0.012−0.064 × sex + 0.024 × day of measurement post‐delivery−0.150 × weight (kg) + 0.055 × weight (kg)^2^ + 0.046 × ethnicity + 0.020 × sum of three skin‐fold thicknesses (triceps, sub scapular, and thigh) [21]. The serum cord leptin (μg/L) to fat mass (kg) ratio was calculated for each infant, and the cohort was classified into top (TT), middle (MT), and low (LT) tertiles across and within sexes. Parametric data were reported as mean and standard deviations, nonparametric data as median and interquartile ranges, and categorical data as percentages. A one‐way repeated measures ANOVA was used to compare maternal leptin across gestation. Data were compared between the TT and LT groups using a chi‐square test, independent *t*‐test, or Mann–Whitney U test, as appropriate. A binary logistic regression was used to test the association between tertiles and pregnancy outcomes adjusting for maternal age, pre‐pregnancy BMI, systolic and diastolic blood pressures, history of chronic hypertension, lifestyle interventions, gestational weight gain, and GDM status. The association of leptin‐to‐fat mass ratio with childhood BMI *z*‐scores at different ages was assessed with simple linear regression analysis. A linear mixed effects model was also used to test the association of tertiles with changes in childhood BMI *z*‐score over time with fixed effects for tertiles, time, sex, tertiles × time, tertiles × sex, gestational weight gain, and lifestyle intervention, adjusting for birth weight *z*‐score. A sensitivity analysis was undertaken using leptin as a continuous variable. The association of the absolute concentration of leptin with adverse pregnancy outcomes was tested using logistic regression, adjusting for fat mass, sex, maternal age, pre‐pregnancy BMI, systolic and diastolic blood pressures, history of chronic hypertension, lifestyle interventions, gestational weight gain, and GDM status. Simple linear regression was used to test its association with childhood BMI *z*‐scores. The analyses were then repeated by using the leptin‐to‐fat mass ratio as a predictor variable and by removing fat mass from the covariates. In addition, an exploratory analysis was performed using maternal adipokines to examine the association of adiponectin and adiponectin‐to‐leptin ratio, measured at 35–37 weeks, with childhood BMI *z*‐score. A *p*‐value of < 0.05 was considered a significant result. All analyses were performed using IBM SPSS Statistics for Macintosh, Version 28.0.

## 3 Results

Of the 1045 DALI participants, 323 (30.9%) had both offspring follow up and umbilical cord data available for analysis. The characteristics of the study population are presented in Table 1. The mean maternal pre‐pregnancy BMI was 33.7 ± 4.2 kg/m^2^, and the majority (83%) of women were of European ethnicity. The maternal leptin concentrations increased from early to late gestation but declined peripartum (*p* = 0.03). The median (IQR) cord leptin concentration was 8.3 (4.5–13.6) ng/mL. The absolute concentration of cord leptin was positively associated with birthweight *z*‐scores (unstandardized coefficient (B) with 95% Confidence interval (CI): 0.023 (0.013–0.033)) and neonatal fat mass percentage (0.110 (0.072–0.148)).

**TABLE 1:** Characteristics of the study population.

Variables	*N*	Mean ± SD/*n* (%)/Median (IQR)
Maternal age (years)	323	32.0 ± 5.2
Pre‐pregnancy BMI (kg/m^2^)	323	33.7 ± 4.2
BMI in early pregnancy (kg/m^2^)	323	34.4 ± 4.3
Ethnicity: European	323	269 (83.3)
OGTT in early pregnancy (mmol/L)		
Fasting glucose	317	4.6 ± 0.4
1 h glucose	304	6.8 ± 1.6
2‐h glucose	305	5.9 ± 1.2
Gestation in early pregnancy (weeks)	323	15.0 ± 2.3
Systolic blood pressure (mmHg)	322	116 ± 10
Diastolic blood pressure (mmHg)	322	73 ± 8
Maternal Leptin in early pregnancy (ng/mL)	302	37.9 ± 17.4
Maternal Leptin at 24–28 weeks (ng/mL)	293	38.2 ± 18.4
Maternal Leptin at 35–37 weeks (ng/mL)	264	38.1 ± 19.1
Maternal Leptin at peripartum (ng/mL)	222	33.9 ± 18.1
Neonate: Gender		
Male	168	52.0
Female	155	48.0
Higher Education	323	196 (60.7)
Marital status: with partner	323	307 (95.0)
Primipara	323	156 (48.3)
Employment status: Working	323	276 (85.4)
Family history of diabetes	323	75 (23.2)
GDM in pregnancy	293	95 (32.4)
Umbilical cord leptin (ng/mL)	323	8.3 (4.5–13.6)
Fetal fat mass (kg)	323	0.4 ± 0.2
Cord leptin fat mass ratio	323	21.1 (12.2–34.8)

Abbreviations: BMI, body mass index; GDM, gestational diabetes mellitus; OGTT, oral glucose tolerance test.

### 3.1 Comparison Between Top and Low Tertiles

Female infants made up 58% of the TT group, whereas male infants made up 66% of the LT group (*p* < 0.001). The maternal characteristics and neonatal outcomes are compared in Table 2. The TT group had significantly higher maternal leptin concentrations throughout gestation and increased gestational weight gain, as well as lower adiponectin‐to‐leptin ratio in the late gestation and reduced vitamin D3 during peripartum. No pregnancy outcomes were consistently different between TT and LT, although caesarean delivery (30.6% vs. 38.0%, *p* = 0.32) was statistically different after adjustment (adjusted odds ratio (95% CI), 0.43 (0.21–0.89)) (Table 3). In comparing the neonatal biomarkers, the TT group had significantly higher cord leptin and cord C‐peptide concentrations and C‐peptide‐to‐glucose ratio, whereas their birth weight *z*‐score and fat mass percentage were similar to those of the LT group (Table 2).

**TABLE 2:** Maternal characteristics and neonatal outcomes stratified by cord leptin fat mass ratio tertiles.

Variables	Low tertile (*n* = 108)		Top tertile (*n* = 108)		*p*
	*n*	Mean ± SD/*n* (%)/median (IQR)	*n*	Mean ± SD/*n* (%)/median (IQR)	
*Maternal characteristics*					
Maternal age (years)	108	32.0 ± 5.3	108	32.1 ± 5.0	0.95
Maternal pre pregnancy BMI (kg/m^2^)	108	33.2 ± 3.4	108	34.0 ± 4.2	0.12
Maternal leptin in early pregnancy (pg/mL)	101	30.2 (22.5–40.1)	104	37.9 (29.6–46.9)	< 0.001
Maternal leptin at 24–28 weeks (pg/mL)	99	29.5 (22.1–39.9)	99	39.2 (28.3–50.5)	< 0.001
Maternal leptin at 35–37 weeks (pg/mL)	88	29.9 (19.2–38.2)	91	41.7 (28.5–60.2)	< 0.001
Maternal leptin at peripartum (pg/mL)	65	24.3 (16.5–34.3)	91	37.2 (24.3–50.2)	< 0.001
Adiponectin at 35–37 weeks (ng/mL)	55	3417 (2489–5194)	44	3077 (2102–4352)	0.21
Adiponectin: leptin ratio 35–37 weeks	52	122.2 (64.5–234.7)	43	80.4 (43.3–103.0)	0.001
Maternal systolic blood pressure in early pregnancy (mm Hg)	107	115.6 ± 9.9	108	117.2 ± 11.1	0.25
Maternal diastolic blood pressure in early pregnancy (mm Hg)	107	72.7 ± 8.1	108	72.5 ± 8.1	0.88
Neck circumference (cm)	108	36.1 ± 2.2	108	36.5 ± 2.2	0.18
Waist circumference (cm)	108	106.5 ± 9.9	108	107.5 ± 9.8	0.47
Maternal triglycerides (mmol/L)	103	1.4 ± 0.5	104	1.3 ± 0.4	0.16
Free fatty acids (mmol/L)	103	0.6 ± 0.2	104	0.6 ± 0.2	0.46
Gestational diabetes mellitus diagnosis in pregnancy	99	30 (30.3)	96	28 (29.2)	0.88
H/O Polycystic ovarian syndrome	105	8 (7.6)	108	11 (10.2)	0.63
H/O Chronic hypertension	108	12 (11.1)	108	15 (13.9)	0.68
Lifestyle interventions (healthy eating, physical activity or both)	106	77 (72.6)	103	75 (72.8)	1.00
Vitamin D supplementation	106	7 (6.6)	103	9 (8.7)	0.61
25OHVitamin D3 at 35–37 weeks (μg/L)	97	28.3 (19.1–40.4)	90	24.2 (14.3–38.7)	0.21
25OHVitamin D3 during peripartum (μg/L)	99	24.6 (16.3–35.1)	104	19.9 (10.4–32.6)	0.04
*Pregnancy/neonatal outcomes*					
Gestational weight gain (kg)	105	7.2 ± 4.4	100	8.7 ± 4.4	0.02
Weight gain above median (7.7 kg)	105	44 (41.9)	100	56 (56.0)	0.51
Gestational age at birth (weeks)	108	39.6 ± 1.3	108	40.0 ± 1.3	0.07
Birthweight (kg)	108	3.6 ± 0.5	108	3.5 ± 0.5	0.35
Birthweight *z*‐score	108	0.3 ± 1.0	108	0.08 ± 0.9	0.12
Neonate sex:	108		108		< 0.001
Male		72 (66.7)		45 (41.7)	
Female		36 (33.3)		63 (58.3)	
Cord serum leptin (μg/L)	108	3.7 (2.2–4.8)	108	16.5 (11.8–21.5)	< 0.001
Cord serum C‐peptide (μg/L)	106	0.56 (0.34–0.79)	108	0.70 (0.48–0.97)	0.004
Cord serum glucose (mmol/L)	101	4.1 (3.4–4.9)	93	4.6 (3.6–5.3)	0.07
C‐peptide to glucose ratio	99	0.14 (0.06–0.18)	94	0.17 (0.11–0.24)	0.003
Neonatal fat mass (kg)	108	0.49 ± 0.2	108	0.46 ± 0.2	0.22
Neonatal fat mass as percentage	108	13.4 ± 3.6	108	12.7 ± 3.7	0.16
Fat free mass as percentage	108	86.6 ± 3.6	108	87.3 ± 3.7	0.16
Small for gestational age	105	7 (6.7)	107	6 (5.6)	0.78
APGAR under 7 at 5 min	105	1 (1.0)	106	0 (0.0)	0.50
Birth weight over 4 kg	108	22 (20.4)	108	18 (16.7)	0.60
Large for gestational age	106	16 (15.1)	108	13 (12.0)	0.55
Pregnancy induced hypertension	106	11 (10.4)	108	21 (19.4)	0.08
Preeclampsia	107	3 (2.8)	108	1 (0.9)	0.37
Preterm birth	108	2 (1.9)	108	1 (0.9)	1.00
Caesarean delivery	108	41 (38.0)	108	33 (30.6)	0.32
Hyperbilirubinemia	98	5 (5.1)	100	1 (1.0)	0.12
Neonatal intensive care unit stay	101	12 (11.9)	94	3 (3.2)	0.03

*Note:* Data were compared between low and top tertile groups.

Low tertile = leptin to fat mass ratio ≤ 12.74; top tertile = leptin to fat mass ratio ≥ 24.54.

**TABLE 3:** Risk of pregnancy outcomes in top leptin‐to‐fat mass ratio tertile group compared to lowest tertile.

Pregnancy outcomes	Odds ratio (95% CI)	Adjusted odds ratio (95% CI)
Small for gestational age	0.83 (0.27–2.54)	1.20 (0.30–4.82)
APGAR under 7 at 5 min	0.19 (0.02–1.64)	Not estimable
Birth weight over 4 kg	0.78 (0.39–1.56)	0.77 (0.36–1.65)
Large for gestational age	0.77 (0.35–1.69)	0.78 (0.33–1.85)
Pregnancy induced hypertension	2.09 (0.95–4.57)	2.59 (0.87–7.67)
Preeclampsia	0.32 (0.03–3.17)	Not estimable
Preterm birth	0.49 (0.04–5.55)	Not estimable
Caesarean delivery	0.72 (0.41–1.26)	0.43 (0.21–0.89)
Hyperbilirubinemia	0.19 (0.02–1.64)	0.20 (0.02–1.99)
Neonatal intensive care unit stay	0.25 (0.07–0.90)	0.27 (0.06–1.19)

*Note:* Adjusted for maternal age, pre‐pregnancy BMI, systolic and diastolic blood pressures, history of chronic hypertension, lifestyle interventions, gestational weight gain, and GDM status.

Low tertile = leptin to fat mass ratio ≤ 12.74; top tertile = leptin to fat mass ratio ≥ 24.54; Comparison = Top tertile group versus low tertile group.

### 3.2 Comparison by Sex

The cord leptin concentration was significantly higher in female infants than in male infants (μg/L) (10.6 (5.7–16.3) vs. 6.7 (4.0–11.2), *p* < 0.001). In terms of body composition, females had lower birth weight (kg) (3.5 ± 0.5 vs. 3.6 ± 0.5, *p* = 0.003) but higher neonatal fat mass percentage (14.2 ± 3.3 vs. 12.4 ± 3.7, *p* < 0.001) compared to males. The leptin‐to‐fat mass ratio was higher in females (median (IQR): 21.4 (13.4, 30.99) vs. 15.5 (8.3, 25.9), *p* < 0.001), while cord C‐peptide and cord glucose concentrations and C‐peptide‐to‐glucose ratio were similar across both sexes (females vs. males: cord C‐peptide, 0.7 (0.4–0.9) vs. 0.7 (0.4–0.8) μg/L, *p* = 0.32, cord glucose, 4.4 (3.5–5.3) vs. 4.2 (3.4–5.1) mmol/L, *p* = 0.30, C‐peptide‐to‐glucose ratio, 0.14 (0.08–0.23) vs. 0.15 (0.09–0.20), *p* = 0.55).

Table 4 presents the differences between tertiles within sexes. Among males, cord C‐peptide and C‐peptide‐to‐glucose ratio were significantly higher in the TT than in the LT group, whereas vitamin D3 during peripartum, birth weight, and fat mass percentage were significantly lower in the TT. Among females, there were no significant differences in cord C‐peptide or C‐peptide‐to‐glucose ratio between the TT and LT groups. The TT had lower maternal adiponectin‐to‐leptin ratio than the LT, while the LT group had lower gestational age at delivery than the TT group (39.1 ± 1.5 vs. 40.0 ± 1.3 weeks of gestation, *p* ≤ 0.001). Hyperbilirubinemia was present only in the LT group (11.4%, vs. 0%, *p* = 0.02).

**TABLE 4:** Comparison of outcomes between leptin‐to‐fat mass ratio tertiles stratified by sex.

Variables	Male (*N* = 168)					Female (*N* = 155)				
	Low tertile (*n* = 56)		Top tertile (*n* = 56)		*p*	Low tertile (*n* = 52)		Top tertile (*n* = 52)		*p*
	*n*	Mean ± SD/*n* (%)/median (IQR)	*n*	Mean ± SD/*n* (%)/median (IQR)		*n*	Mean ± SD/*n* (%)/median (IQR)	*n*	Mean ± SD/*n* (%)/median (IQR)	
Maternal leptin < 20 weeks	53	32.8 (23.2–41.2)	55	38.3 (28.0–48.1)	0.02	45	31.9 (23.4–41.1)	49	34.3 (26.7–45.8)	0.12
Leptin at 24–28 weeks (pg/mL)	53	29.8 (21.4–38.5)	51	38.5 (30.7–47.6)	< 0.001	44	31.0 (23.3–42.3)	49	36.5 (26.9–51.3)	0.11
Leptin at 35–37 weeks (pg/mL)	45	29.6 (20.4–38.7)	42	41.0 (29.9–59.4)	< 0.001	40	29.8 (19.4–42.7)	47	38.8 (26.1–62.9)	0.02
Leptin at peripartum (pg/mL)	29	24.3 (13.7–34.0)	48	40.0 (30.5–55.9)	< 0.001	34	24.4 (16.6–37.1)	42	34.9 (19.2–46.5)	0.09
GDM in pregnancy	52	10 (19.2)	48	14 (29.2)	0.35	43	19 (44.2)	49	15 (30.6)	0.20
Adiponectin at 35–37 weeks (ng/mL)	28	3450 (2624–4612)	20	3837 (2887–5957)	0.29	26	3510 (2523–6014)	23	2523 (2088–3628)	0.054
Adiponectin: leptin ratio at 35–37 weeks	25	92.1 (57.8–231.6)	17	84.8 (46.8–122.3)	0.24	26	151.3 (84.3–263.5)	23	78.7 (43.3–100.5)	0.001
25OHVitamin D3 at 35–37 weeks (μg/L)	50	28.4 (19.0–42.3)	45	23.8 (14.2–35.8)	0.10	44	26.0 (18.6–38.9)	45	24.2 (14.0–40.4)	0.79
25OHVitamin D3 during peripartum (μg/L)	53	25.6 (15.9–35.1)	52	18.0 (10.5–28.5)	0.008	47	23.8 (14.0–35.3)	51	24.8 (9.8–32.9)	0.61
Gestational weight gain (kg)	54	7.3 ± 4.1	50	9.0 ± 4.9	0.059	50	20.4 ± 2.6	51	20.9 ± 2.3	0.40
Gestation at delivery (weeks)	56	39.9 ± 1.3	56	39.8 ± 1.3	0.72	52	39.1 ± 1.5	52	40.0 ± 1.3	≤ 0.001
Birthweight (kg)	56	3.7 ± 0.4	56	3.5 ± 0.5	0.045	52	3.4 ± 0.5	52	3.5 ± 0.4	0.12
Birthweight *z*‐core	56	0.24 ± 0.84	56	−0.11 ± 0.98	0.047	52	0.24 ± 1.0	52	0.19 ± 0.7	0.77
Cord serum leptin (μg/L)	56	2.9 (2.0–4.3)	56	12.9 (9.0–20.3)	< 0.001	52	4.5 (3.0–6.7)	52	17.7 (13.1–23.9)	< 0.001
Cord serum C‐peptide (μg/L)	55	0.5 (0.1–0.7)	56	0.7 (0.5–1.0)	< 0.001	51	0.7 (0.5–0.9)	52	0.7 (0.5–1.0)	0.49
Cord serum glucose (mmol/L)	54	4.1 (3.4–4.9)	50	4.6 (3.7–5.6)	0.11	45	4.6 (3.7–5.4)	44	4.6 (3.6–5.4)	0.85
C‐peptide to glucose ratio	53	0.1 (0.02–0.2)	50	0.2 (0.1–0.2)	< 0.001	45	0.15 (0.06–0.19)	44	0.15 (0.10–0.25)	0.15
Neonatal fat mass (kg)	56	0.5 ± 0.2	56	0.4 ± 0.2	0.02	52	0.5 ± 0.2	52	0.5 ± 0.2	0.99
Neonatal fat mass as %	56	13.0 ± 3.6	56	11.1 ± 3.9	0.008	52	14.2 ± 3.8	52	13.7 ± 3.0	0.51
Fat free mass as %	56	87.0 ± 3.6	56	88.8 ± 3.9	0.008	52	85.8 ± 3.8	52	86.3 ± 3.0	0.51
Small for gestational age	55	2 (3.6)	56	4 (7.1)	0.68	50	6 (12.0)	52	1 (1.9)	0.057
Birth weight above 4 kg	56	12 (21.4)	56	12 (21.4)	1.00	52	7 (13.5)	52	6 (11.5)	1.00
Large for gestational age	55	7 (12.7)	56	8 (14.3)	1.00	51	7 (13.7)	52	5 (9.6)	0.55
PIH	54	4 (7.4)	56	7 (12.5)	0.53	52	5 (9.6)	52	10 (19.2)	0.26
Preterm birth	56	0 (0.0)	56	1 (1.8)	1.00	52	4 (7.7)	52	0 (0.0)	0.12
Caesarean delivery	56	25 (44.6)	56	19 (33.9)	0.33	52	18 (34.6)	52	15 (28.8)	0.67
Hyperbilirubinemia	52	2 (3.8)	51	1 (2.0)	1.00	44	5 (11.4)	49	0 (0.0)	0.02
NICU stay	52	7 (13.5)	48	2 (4.2)	0.16	48	4 (8.3)	47	1 (2.1)	0.36

*Note:* Data were compared between low and top tertile groups.

Male: Low tertile = leptin to fat mass ratio ≤ 10.28; top tertile = leptin to fat mass ratio ≥ 20.83.

Female: Low tertile = leptin to fat mass ratio ≤ 15.93; top tertile = leptin to fat mass ratio ≥ 26.46.

Abbreviations: GDM, gestational diabetes mellitus; PIH, pregnancy induced hypertension; NICU, neonatal intensive care unit.

### 3.3 Association Between Leptin Tertiles and Childhood BMI

A comparison of childhood BMI *z*‐scores between the TT and LT groups showed that scores were similar from age 1 through age 5 (Table S1). Among males, the TT had lower BMI *z*‐scores (except at age 4) with significant difference found only at age 1 year (Table S2), whereas among females, the BMI *z*‐scores were similar between the TT and LT groups (Table S2). In the mixed effects model, the tertiles were not associated with changes in childhood BMI *z*‐scores over time (*p* = 0.81). The estimated marginal means (std. error) for the TT and LT groups based on the fitted model were 0.775 (0.1) and 0.822 (0.14), and the difference between the groups was not statistically significant (0.047(0.19), *p* = 0.81). The birthweight *z*‐score was not significantly associated with BMI *z*‐score change (*p* = 0.15), and no effects were noted for infant sex or the interaction between tertiles and sex (*p* = 0.58, and *p* = 0.12, respectively).

### 3.4 Sensitivity Analyses Using Leptin as a Continuous Variable

The absolute concentration of cord leptin was not associated with adverse pregnancy outcomes (Table S3) or high BMI *z*‐scores in early childhood (unstandardized beta coefficient): age 1, 0.010, *p* = 0.44, age 2: −0.003, *p* = 0.84, age 3: −0.027, *p* = 0.09, age 4: 0.021, *p* = 0.08, age 5: −0.015, *p* = 0.39. Similar findings were obtained when the analyses were repeated using leptin‐to‐fat mass ratio. There were no associations with adverse pregnancy outcomes (Table S4) or high BMI *z*‐scores (unstandardized beta coefficient: age 1, −0.004, *p* = 0.28; age 2, −0.006, *p* = 0.08; age 3, −0.001, *p* = 0.75; age 4, −0.002, *p* = 0.59; age 5, −0.008, *p* = 0.07).

In the exploratory analysis, neither maternal adiponectin nor the adiponectin‐to‐leptin ratio showed a significant correlation with childhood BMI *z*‐score at any age (Table S5).

## 4 Discussion

In this study, we examined the relationship between relative cord hyperleptinemia and offspring outcomes at birth and in early childhood, using leptin‐to‐fat mass ratio tertiles. We explored the association across and within sexes. We found that relative cord hyperleptinemia in pregnant women with overweight or obesity was associated with a higher cord C‐peptide concentration and higher C‐peptide‐to‐glucose ratio at birth, with lower BMI *z*‐score in the first year of age for males but not for females. There was no difference between the tertiles in major adverse pregnancy outcomes in the adjusted model.

Previous research has shown that maternal leptin concentrations increase as gestation advances and return to pre‐pregnancy concentrations after birth [22, 23, 24]. This pattern is consistent with our results. However, previous research has also shown that among mothers with obesity who enter pregnancy with elevated leptin concentrations, the amount of leptin secreted per unit mass of adipose or placental tissue is lower than in mothers with normal weight, partially due to lower gestational weight gain [23]. Although a significant positive correlation has been reported between maternal and fetal leptin [25], there is no leptin transport across the placenta due to its high molecular weight [26]. It is estimated that 95% of placental leptin is released into the maternal circulation and only 5% into the fetal circulation [27], thereby establishing fetal fat mass as the major origin of fetal leptin [27]. Our study showed a positive correlation between umbilical cord leptin and neonatal adiposity. In a meta‐analysis of 22 studies, cord leptin concentration was positively correlated with neonatal adiposity (*r* = 0.487 (0.444–0.531)) [9], reflecting its fetal source. In a secondary analysis of the Hyperglycemia and Adverse Pregnancy Outcome (HAPO) study and the HAPO Follow‐Up Study (HAPOFUS), a 1 SD increase in log‐cord leptin concentration was associated with a 207 g increase in birthweight, a 78 g increase in body fat mass, a 1.6% increase in body fat percentage, and a 1.2 mm increase in neonatal sum of skin‐folds, after adjusting for relevant confounders [8].

Previous offspring studies on cord leptin have shown a negative relationship between higher cord leptin concentrations and infant weight gain in the first 6 months of age and adiposity at 3 years of age [9, 10, 28, 29, 30]. In our sensitivity analysis, although we noticed a negative relationship between cord leptin and BMI at ages 2, 3, and 5 years, this did not reach statistical significance, possibly due to low sample sizes. Meanwhile, studies in older children reported inconsistent findings [8, 11, 31]. The HAPOFUS study showed a strong positive relationship between cord leptin concentrations and adiposity later in childhood (mean age 11.5 years) [8], but Project Viva found no relationship in mid‐childhood (mean age 7.9 years) or early adolescence (mean age 13.2 years) [31]. In contrast to our hypothesis, our findings suggest that offspring born to mothers with overweight or obesity, who are exposed to a high‐leptin environment in utero are more leptin sensitive in early years, as seen in normal weight pregnancies [30].

Evidence regarding sex differences and cord leptin concentrations is mixed. Some research suggests there is no association with gender, particularly in later years [8, 31]; meanwhile, others have found increased concentrations of cord leptin in female infants compared to male infants [10, 32, 33, 34]. This latter sex difference could be partially caused by the inhibitory effect of androgen on leptin expression in males [34]. In line with prior research, our cohort exhibited significantly higher cord leptin concentrations in female infants and an inverse relationship between relative cord hyperleptinemia and childhood BMI only among male infants. More importantly, male infants with relative cord hyperleptinemia also had significantly higher cord C‐peptide concentrations and C‐peptide‐to‐glucose ratios suggesting possible insulin resistance. There is sex‐specific placental adaptation to the lipotoxic environment in pregnancies affected by obesity [35]. It has been suggested that female placentas are more adaptable to an obesogenic environment, marked by decreased fatty acid oxidation and increased fatty acid esterification and placental inflammation [35]. However, similar to other studies, our study also found more neonatal adiposity in female infants vs. male infants. Another finding in this study is the significant presence of hyperbilirubinemia among female infants who belonged to the LT group. A direct relationship between bilirubin and leptin concentrations is uncertain, however, we assume that a lower gestational age at delivery and a higher number of preterm births in the LT group could have contributed to hyperbilirubinemia in this group. Further research is needed to determine whether this association between hyperbilirubinemia and leptin persists in different populations or whether it was unique to the DALI cohort.

Our study is the first to utilize the leptin‐to‐fat mass ratio to predict offspring outcomes. In previous studies, researchers have used cord leptin concentrations or adiponectin/leptin ratio [36]. A notable strength of this study is its population. Because the DALI trial took place across Europe, we could select from a large pool of participants for the secondary analysis. In addition, the longitudinal design of the study allowed us to observe the changes in BMI across early childhood to determine whether there was any significant association. However, the study also has several limitations. First, childhood height and weight were collected by surveying study participants, which could have led to response bias. Second, the data were self‐reported and not objectively measured by the research team; therefore, the validity and reliability of the data could not be ascertained. Another potential limitation of this study is the homogeneity of the study participants. Because of the locations where data were collected, the study population consisted of mainly European women. Only 16.7% of the population was non‐European. Additionally, although our study tracked childhood BMIs from ages 1 through 5 as a marker of adiposity, only a small sample of data was available to analyse compared to the original 1045 infants included. Follow‐up data were limited because of the lack of follow‐up survey responses from participants in the original study. Finally, because the DALI trial included only mothers with overweight or obesity, the lack of a control group of pregnant women with normal weight limited our ability to determine the true effect of cord hyperleptinemia on childhood BMI. Future research should include a more diverse participant population and a larger sample size to more consistent with an average population. It is also important to recognize that the data presented in this study needs to be interpreted carefully because an arbitrary criterion has been used to define leptin resistance. The clinical application of the research has yet to be proven.

## 5 Conclusion

In conclusion, in this secondary analysis of the DALI trial, we found that relative cord hyperleptinemia in women with overweight or obesity was inversely associated with offspring adiposity at birth and BMI *z*‐scores in infancy among males. This may imply that despite being exposed to high concentrations of leptin in utero, male infants still retain leptin sensitivity after birth and in early childhood. Further studies are required to evaluate the implications of these findings for future metabolic health.

## Author Contributions

J.I. undertook the statistical analyses, interpreted the data, and drafted the manuscript. S.C. contributed to the statistical analyses and drafting of the manuscript. D.S. conceived the project, interpreted the data, reviewed and edited the draft and provided critical input to the manuscript. D.S., R.C., R.D., A.V.A., A.L., E.W., A.K., F.D., P.D., E.R.M., D.J.H. and F.S. designed and executed the DALI study and provided input for the interpretation of the results. D.S. was the study coordinator. J.M.A., S.G., M.G.D., A.B., J.H., A.Z., D.M.J., L.T.A., J.G.M.J. contributed to the execution of the study and provided input for the interpretation of the results. G.D. designed and executed the study and provided input for the interpretation of the results. He was the principal investigator of the study. M.N.M.P. designed and executed the study and provided input for the interpretation of the results. She was the sponsor of the study. All authors read and approved the final manuscript. J.I. is the guarantor of this work and takes full responsibility for the contents of this article.

## Funding

The project described received funding from the European Community’s Seventh Framework Programme (FP7/2007‐ 2013) under grant agreement no. 242187. In the Netherlands, additional funding was provided by the Netherlands Organisation for Health Research and Development (ZonMw) (grant 200310013). In Poland, additional funding was obtained from Polish Ministry of Science (grants 2203/7,PR/2011/2). In Denmark, additional funding was provided by Odense University Free Research Fund. In the United Kingdom, the DALI team acknowledge the support received from the NIHR Clinical Research Network: Eastern, especially the local diabetes clinical and research teams based in Cambridge. In Spain, additional funding was provided by CAIBER 1527‐B‐226. The funders had no role in any aspect of the study beyond funding.

## Conflicts of Interest

The authors declare no conflicts of interest.

## Supporting information


**Table S1:** Comparison of BMI *z*‐score trajectories between low and top tertile groups.
**Table S2:** Comparison of BMI *z*‐score trajectories between low and top tertile groups by sex.
**Table S3:** Association between absolute concentration of cord leptin and adverse pregnancy outcomes.
**Table S4:** Association between cord leptin‐to‐fat mass ratio and adverse pregnancy outcomes.
**Table S5:** Correlation between adipokine markers and childhood body mass index *z‐*scores.

## Data Availability

The data that support the findings of this study are available from the corresponding author upon reasonable request.
