# Supplementary material for: Association Between Relative Cord Hyperleptinemia and Offspring Outcomes in Pregnant Women With Overweight or Obesity
Source: Pediatr Obes. 2026 Aug 21;21(8):e70144. doi: 10.1111/ijpo.70144 (PMC13498720; doi:10.1111/ijpo.70144)
Supplement: Supplementary file 1 [file IJPO-21-e70144-s001.pdf]

Association between relative cord hyperleptinemia and offspring outcomes in pregnant women with overweight or obesity

Authors:

Jincy Immanuel[1,2,] Stephy Chirayath[2], Jürgen Harreiter[3,4], Gernot Desoye[5], Rosa Corcoy[6,7,8] , Juan M. Adelantado[9], Roland Devlieger[9,10], Annunziata Lapolla[11], Maria G. Dalfra[11], Alessandra Bertolotto[12], Ewa Wender–Ozegowska[13], Agnieszka Zawiejska[13], Fidelma P. Dunne[14], Peter Damm[15,16], Elisabeth R. Mathiesen[15,16], Dorte M. Jensen[17,18,19], Lise Lotte T. Andersen[18,19], Mette H. Tanvig[18,19], David J. Hill[20], Judith G.M. Jelsma[21], Alexandra Kautzky–Willer[3], Sander Galjaard[9,10,22], Frank J. Snoek[23], Mireille N.M. van Poppel[21,24] David Simmons [1]

Affiliations:

[1] School of Medicine, Western Sydney University, Sydney, Australia

[2] College of Nursing, Texas Woman's University, Texas, USA

[3] Department of Medicine III, Division of Endocrinology, Gender Medicine Unit Medical University of Vienna, Vienna, Austria

[4] Department of Medicine and Palliative Care, Landesklinikum Scheibbs, Scheibbs, Austria.

[5] Department of Obstetrics and Gynecology, Medizinische Universitaet Graz, Graz, Austria

[6] Department of Medicine, Universitat Autònoma de Barcelona, Barcelona, Spain

[7] Institut de Recerca de l´Hospital de la Santa Creu i Sant Pau, Barcelona, Spain

[8] CIBER Bioengineering, Biomaterials and Nanotechnology, Instituto de Salud Carlos III, Madrid, Spain

[9] KU Leuven Department of Development and Regeneration: Pregnancy, Fetus and Neonate, Belgium

[10] Gynaecology and Obstetrics, University Hospitals Leuven, Belgium

[11] Universita Degli Studi di Padova, Padua, Italy

[12] Azienda Ospedaliero–Universitaria Pisana, Pisa, Italy

[13] Department of Reproduction, Poznan University of Medical Sciences, Poland

[14] National University of Ireland, Galway, Ireland

[15] Center for Pregnant Women with Diabetes, Departments of Endocrinology and Obstetrics, Rigshospitalet, Copenhagen, Denmark

[16] Department of Clinical Medicine, University of Copenhagen, Copenhagen, Denmark

[17] Steno Diabetes Center Odense, Odense University Hospital, Odense, Denmark

[18] Department of Gynecology and Obstetrics, Odense University Hospital, Odense, Denmark

[19] Department of Clinical Research, Faculty of Health Science, University of Southern Denmark, Odense, Denmark

[20] Lawson Research Institute, London, Ontario, Canada

[21] Amsterdam UMC, Vrije Universiteit Amsterdam, Department of Public and Occupational Health, Amsterdam Public Health research institute, Amsterdam, The Netherlands

[22] Department of Obstetrics and Gynaecology, Division of Obstetrics and Prenatal Medicine, Erasmus MC, University Medical Centre, Rotterdam, the Netherlands

[23] Amsterdam UMC, Vrije Universiteit Amsterdam, Department of Medical Psychology, Amsterdam, The Netherlands

[24] Department of Human Movement Science, Sport and Health, University of Graz, Graz, Austria

Supplementary Table 1. Comparison of BMI z-score trajectories between low and top tertile groups

Age	Low Tertile (n=108)		Top Tertile (n=108)		p value
	n	Median (IQR)	n	Median (IQR)	
Age 1	28	0.90 (0.05–1.56)	26	0.32 (-0.27 –1.22)	0.18
Age 2	26	1.06 (0.49–1.88)	26	0.61 (-0.12–1.65)	0.16
Age 3	24	0.99 (0.03–1.45)	22	0.95 (0.22–1.49)	0.90
Age 4	20	0.87 (0.08–1.47)	18	0.81 (0.23–1.54)	0.85
Age 5	10	0.71 (0.02–1.10)	8	0.89 (-0.63–1.20)	0.20

Groups were compared using the Mann-Whitney U test.

Supplementary Table 2. Comparison of BMI z score trajectories between low and top tertile groups by sex

BMI assessment	Male (N=168)					Female (N=155)				
	Low Tertile (n=56)		Top Tertile (n=56)		p value	Low Tertile (n=52)		Top Tertile (n=52)		p value
	n	Median (IQR)	n	Median (IQR)		n	Median (IQR)	n	Median (IQR)	
Age 1	15	1.35 (-0.06–1.67_	13	-0.15 (-0.67–0.98)	0.03	13	0.61 (0.02–1.03)	13	0.75 (-0.11–1.46)	0.76
Age 2	15	1.01 (-0.06–2.03)	15	0.34 (-0.98–0.88)	0.10	11	1.28 (-0.33–1.84)	11	0.50 (-0.28–1.94)	0.95
Age 3	16	1.24 (0.32–1.47)	12	0.42 (-0.28–1.51)	0.42	7	0.79 (-0.11–0.98)	12	1.12 (0.42–1.61)	0.34
Age 4	12	0.86 (0.07–1.91)	16	0.97 (0.16–1.80)	0.95	9	0.41 (-0.22–1.58)	4	1.26 (0.62–1.55)	0.50
Age 5	5	1.07 (0.98–3.03)	7	0.77 (-0.22–1.21)	0.11	4	0.18 (-0.12–1.65)	4	1.17 (-0.52–1.57)	1.00

Supplementary Table 3. Association between absolute concentration of cord leptin and adverse pregnancy outcomes

Pregnancy outcomes	Unadjusted model			Adjusted model		
	Nagelkerke R Square	B (SE)	OR (95% CI)	Nagelkerke R Square	B (SE)	Adj OR (95% CI)
Small for gestational age	0.05	-0.097 (0.053)	0.91 (0.82-1.01)	0.05	-0.038 (0.062)	0.96 (0.85-1.09)
APGAR under 7 at 5 mins	0.03	-0.090 (0.118)	0.92 (0.73-1.15)	--	--	Not estimable
Birth weight over 4 kg	0.09	0.059 (0.015)	1.06 (1.03-1.09)	0.72	0.031 (0.022)	1.03 (0.98-1.08)
Large for gestational age	0.10	0.061 (0.015)	1.06 (1.03-1.10)	0.64	0.041 (0.021)	1.04 (0.999-1.09)
Pregnancy induced hypertension	0.01	0.014 (0.014)	1.01 (0.99-1.04)	0.38	0.025 (0.017)	1.03 (0.99-1.06)
Preeclampsia	0.05	-0.135 (0.098)	0.87 (0.72-1.06)	0.61	-0.715 (0.437)	0.49 (0.21-1.15)
Preterm birth	0.02	-0.060 (0.063)	0.094 (0.83-1.07)	0.45	0.073 (0.067)	1.08 (0.94-1.23)
Cesarean delivery	0.02	-0.025 (0.014)	0.98 (0.95-1.00)	0.22	-0.034 (0.019)	0.97 (0.93-1.00)
Hyperbilirubinemia	0.01	-0.040 (0.050)	0.96 (0.87-1.06)	0.26	-0.152 (0.092)	0.86 (0.72-1.03)
Neonatal intensive care unit stay	0.01	-0.034 (0.035)	0.97 (0.90-1.04)	0.10	-0.044 (0.041)	0.96 (0.88-1.04)

Adjusted for fat mass, sex, maternal age, pre-pregnancy BMI, systolic and diastolic blood pressures, history of chronic hypertension, lifestyle interventions, gestational weight gain, and GDM status.

Supplementary Table 4. Association between cord leptin-to-fat mass ratio and adverse pregnancy outcomes

Pregnancy outcomes	Unadjusted model			Adjusted model		
	Nagelkerke R Square	B (SE)	OR (95% CI)	Nagelkerke R Square	B (SE)	Adj OR (95% CI)
Small for gestational age	0.04	0.008 (0.004)	1.01 (1.00-1.02)	0.18	-0.011 (0.019)	0.99 (0.95-1.03)
APGAR under 7 at 5 mins	0.02	-0.031 (0.052)	0.97 (0.88-1.07)	0.71	0.000 (0.083)	1.00 (0.85-1.18)
Birth weight over 4 kg	0.001	-0.002 (0.005)	0.998 (0.99-1.01)	0.10	-0.002 (0.007)	0.998 (0.98-1.01)
Large for gestational age	0.000	0.000 (0.004)	1.00 (0.99-1.01)	0.14	0.003 (0.008)	1.003 (0.99-1.02)
Pregnancy induced hypertension	0.04	0.014 (0.007)	1.01 (1.00-1.03)	0.36	0.015 (0.008)	1.02 (1.00-1.03)
Preeclampsia	0.02	-0.035 (0.038)	0.97 (0.90-1.04)	0.55	-0.189 (0.111)	0.83 (0.67-1.03)
Preterm birth	0.12	0.014 (0.010)	1.01 (0.996-1.03)	0.23	0.025 (0.016)	1.03 (0.99-1.06)
Cesarean delivery	0.000	0.001 (0.003)	1.001 (0.995-1.01)	0.21	-0.012 (0.008)	0.99 (0.97-1.004)
Hyperbilirubinemia	0.04	-0.049 (0.033)	0.95 (0.89-1.02)	0.24	-0.065 (0.043)	0.94 (0.86-1.02)
Neonatal intensive care unit stay	0.007	-0.011 (0.016)	0.99 (0.96-1.02)	0.08	-0.006 (0.02)	0.99 (0.97-1.02)

Adjusted for sex, maternal age, pre-pregnancy BMI, systolic and diastolic blood pressures, history of chronic hypertension, lifestyle interventions, gestational weight gain, and GDM status

Supplementary Table 5. Correlation between adipokine markers and childhood body mass index z scores

BMI assessment	Adiponectin at 35-37 weeks			Adiponectin Leptin ratio at 35-37 weeks		
	n	r	p value	n	rs	p value
Age 1	44	-0.228	0.14	40	0.034	0.83
Age 2	43	-0.129	0.41	39	0.134	0.42
Age 3	41	-0.084	0.60	38	-0.249	0.13
Age 4	31	0.139	0.46	28	0.195	0.32
Age 5	20	-0.306	0.19	18	-0.292	0.24

r= Pearson correlation coefficient; rs = Spearman’s rho
